# [Retracted] Protection of Luteolin-7-O-glucoside against apoptosis induced by hypoxia/reoxygenation through the MAPK pathways in H9c2 cells

**DOI:** 10.3892/mmr.2025.13591

**Published:** 2025-06-10

**Authors:** Shenjie Chen, Bingsheng Yang, Yifei Xu, Yiqing Rong, Yuangang Qiu

Mol Med Rep 17: 7156-7162, 2018; DOI: 10.3892/mmr.2018.8774

Following the publication of the above paper, it was drawn to the Editor's attention by a concerned reader that, regarding the lower left quadrants of certain of the flow cytometric plots shown in Fig. 3A on p. 7159, these appeared to show similar groupings of dots, which would not have been anticipated if these experiments had been performed discretely under different experimental conditions, suggesting a fundamental flaw either in the way in which these experiments were performed or in how the results were outputted. Moreover, these data were also strikingly similar in appearance to data that had been submitted to the same journal around the same time in an article written by different authors at a different research institute.

The Editor of *Molecular Medicine Reports* has decided that this paper should be retracted from the Journal on account of a lack of confidence in the presentation and the originality of the data. The authors were asked for an explanation to account for these concerns, but the Editorial Office did not receive a reply. The Editor apologizes to the readership for any inconvenience caused.

